# System-wide analyses of the fission yeast poly(A)^+^ RNA interactome reveal insights into organization and function of RNA–protein complexes

**DOI:** 10.1101/gr.257006.119

**Published:** 2020-07

**Authors:** Cornelia Kilchert, Tea Kecman, Emily Priest, Svenja Hester, Ebru Aydin, Krzysztof Kus, Oliver Rossbach, Alfredo Castello, Shabaz Mohammed, Lidia Vasiljeva

**Affiliations:** 1 Institut für Biochemie, Justus-Liebig-Universität Gießen, 35392 Gießen, Germany;; 2 Department of Biochemistry, University of Oxford, Oxford, OX1 3QU, United Kingdom;; 3 Department of Chemistry, University of Oxford, Chemistry Research Laboratory, Oxford, OX1 3TA, United Kingdom

## Abstract

Large RNA-binding complexes play a central role in gene expression and orchestrate production, function, and turnover of mRNAs. The accuracy and dynamics of RNA–protein interactions within these molecular machines are essential for their function and are mediated by RNA-binding proteins (RBPs). Here, we show that fission yeast whole-cell poly(A)^+^ RNA–protein crosslinking data provide information on the organization of RNA–protein complexes. To evaluate the relative enrichment of cellular RBPs on poly(A)^+^ RNA, we combine poly(A)^+^ RNA interactome capture with a whole-cell extract normalization procedure. This approach yields estimates of in vivo RNA-binding activities that identify subunits within multiprotein complexes that directly contact RNA. As validation, we trace RNA interactions of different functional modules of the 3′ end processing machinery and reveal additional contacts. Extending our analysis to different mutants of the RNA exosome complex, we explore how substrate channeling through the complex is affected by mutation. Our data highlight the central role of the RNA helicase Mtl1 in regulation of the complex and provide insights into how different components contribute to engagement of the complex with substrate RNA. In addition, we characterize RNA-binding activities of novel RBPs that have been recurrently detected in the RNA interactomes of multiple species. We find that many of these, including cyclophilins and thioredoxins, are substoichiometric RNA interactors in vivo. Because RBPomes show very good overall agreement between species, we propose that the RNA-binding characteristics we observe in fission yeast are likely to apply to related proteins in higher eukaryotes as well.

A major challenge in the field of RNA regulation is to understand how large multi-subunit complexes interact with RNA. The cleavage and polyadenylation machinery, for example, is a megadalton assembly that processes the 3′ ends of RNAs transcribed by RNA polymerase II (Pol II). It consists of the cleavage and polyadenylation factor (CPF) and the accessory cleavage factors IA and IB (CFIA and CFIB). It recognizes polyadenylation sites (PAS) and auxiliary regulatory sequences, and it induces cleavage of the nascent transcript followed by polyadenylation of the 3′ end (Wahle and Rüegsegger 1999; Zhao et al. 1999; Proudfoot 2011; Kumar et al. 2019; Thore and Fribourg 2019). Studies of the isolated protein complex revealed an organization into functionally divergent modules (Casañal et al. 2017; Hill et al. 2019). In addition, reconstitution of individual modules with the PAS or auxiliary sequence elements has identified protein–RNA contacts that mediate recognition of consensus motifs (Pancevac et al. 2010; Barnwal et al. 2012; Schönemann et al. 2014; Clerici et al. 2017, 2018; Sun et al. 2018). Despite this progress, it is not clear how interactions between the pre-mRNA and the full complex beyond consensus motif recognition help to ensure accurate PAS selection.

Crosslinking of recombinant in vitro reconstituted ribonucleoprotein complexes (RNPs) has been a powerful tool to identify proteins that interact with RNA in the context of large RNPs (Schmidt et al. 2012). However, this method requires protein complex purification, which in the case of large machineries is not a trivial task. Moreover, reconstituted complexes may not behave as in the cellular environment, where they engage in active and dynamic interactions with RNA. These challenges prevent us from understanding how essential machineries involved in various aspects of RNA regulation function in vivo.

In recent years, RNA–protein interactions have been systematically studied using in vivo, system-wide approaches. Crosslinking and immunoprecipitation (CLIP) has become the method of choice to identify RNAs bound by an RBP of interest. It can do so with nucleotide resolution (Ule et al. 2003; Hafner et al. 2010; König et al. 2010). Despite its power, CLIP is subject to one limitation: As a technique that is inherently single-protein, signal strength for one RBP cannot be directly compared to others to assess protein–RNA association in relative terms. Conversely, the development of RNA interactome capture (RIC), which combines oligo(dT) enrichment of RNA–protein crosslinks with quantitative mass spectrometry (MS), has served to catalog the “RBPome” of all polyadenylated RNAs in different model systems (Baltz et al. 2012; Castello et al. 2012; Kwon et al. 2013; Mitchell et al. 2013; Beckmann et al. 2015; Matia-González et al. 2015; Conrad et al. 2016; Sysoev et al. 2016; Marondedze et al. 2019). Classically, however, RIC quantified RNA–protein crosslinks in absolute terms and disregarded differences in protein abundance.

It was the aim of this study to develop a modified RIC analysis workflow that allows the comparison of in vivo RNA-binding activities of RBPs and enhancement of the applicability of RIC to the study of RNPs. To this end, we determined the RNA interactome of the fission yeast *Schizosaccharomyces pombe* and introduced a whole-cell extract (WCE)-normalization procedure to assess relative enrichment of cellular RBPs. To evaluate performance of the method, we validated RNA-binding behavior of selected RBPs experimentally and applied our analysis to RNA–protein interactions within different multi-subunit RNPs—namely, the 3′ end processing machinery and the RNA exosome complex.

## Results

### *S. pombe* poly(A)^+^ RNA interactome capture

**Figure 1.**
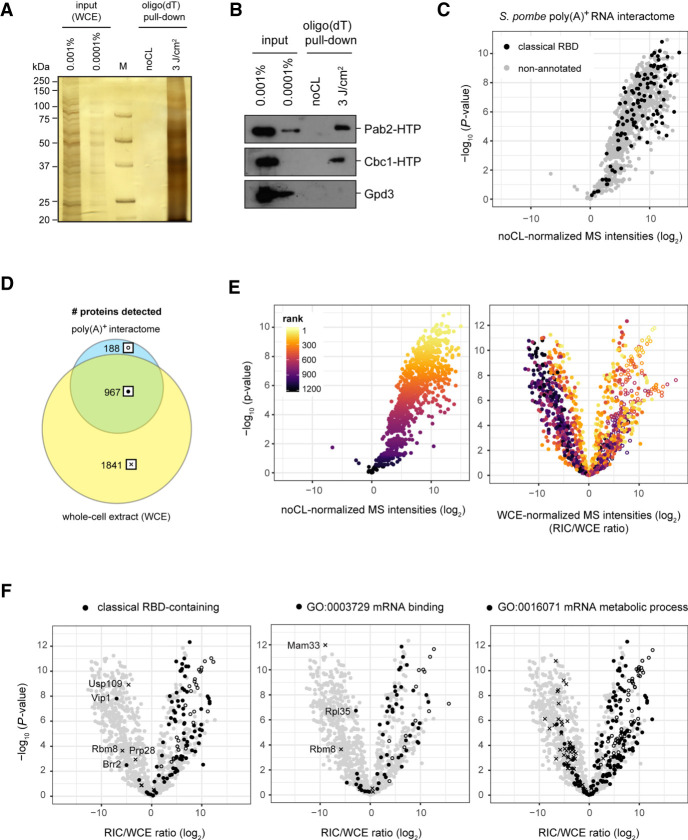
Poly(A)^+^ RNA interactome capture in *S. pombe*. Quality control of a representative RIC experiment using silver staining (*A*) or western blotting (*B*) of input and proteins eluted from oligo(dT) beads. RNA interactors were only recovered if cells were irradiated with UV before harvesting (3 J/cm^2^), but not from the non-crosslinked controls (noCL). Known RBPs such as the poly(A)-binding protein Pab2 or the cap-binding protein Cbc1 were robustly detected in the crosslinked samples; the abundant metabolic enzyme Gpd3 was not. (*C*) Mass spectrometry (MS) analysis of the *S. pombe* poly(A)^+^ RNA interactome. In the volcano plot, *P*-values (−log, moderated Student's *t*-test) are plotted against the fold change of mean MS intensities (log_2_) of proteins recovered from the oligo(dT) pull-downs of UV-crosslinked samples (3 J/cm^2^) (*n* = 6) normalized to noCL controls (*n* = 3). Background values were imputed for proteins without signal in the noCL control. Proteins annotated with a classical RNA-binding domain (RBD) are designated in black. The corresponding data can be found in Supplemental Table S1, and the list of Pfam identifiers used for this analysis are in Supplemental Table S2. (*D*) Overlap of proteins detected in oligo(dT) pull-downs from UV-crosslinked cells (3 J/cm^2^) and the corresponding whole-cell extracts (WCE) (*n* = 6). In all downstream analyses, the indicated symbols are used to designate proteins from a given population: full circles for proteins detected in oligo(dT) pull-down and WCE; empty circles for proteins detected in oligo(dT) pull-downs but not the WCE; crosses for proteins detected in the WCE but not the oligo(dT) pull-downs. (*E*) Impact of normalization method on relative RBP enrichment in the poly(A)^+^ RNA interactome. *P*-values (−log, moderated Student's *t*-test) are plotted against the fold change of mean MS intensities (log_2_) of proteins recovered from the oligo(dT) pull-downs of UV-crosslinked samples (3 J/cm^2^) over either noCL controls (*left*) or the input WCE (RIC/WCE ratios, *right*) (*n* = 6). For WCE normalization (*right*), background values were imputed for proteins without WCE signal (empty circles) and raw MS intensities were normalized to median = 0 before calculating fold-change values. In both panels, individual proteins are colored according to the statistical significance (ranked *P*-values) of protein enrichment in the noCL-normalized interactome. (*F*) Distribution of proteins annotated with a classical RBD (*left*), GO function “mRNA binding” (*middle*), or GO process “mRNA metabolic process” (*right*) in the WCE-normalized RNA interactome designated in black, using symbols to indicate protein populations as in *D*. Background values were imputed for proteins without signal in any given sample.

Fission yeast recapitulates many aspects of mammalian RNA regulation. Yet, no systematic analysis of RBPs had been performed in the model organism. To determine the *S. pombe* poly(A)^+^ RNA interactome, we UV-crosslinked wild-type (WT) cells labeled with 4-thiouracil (4sU). RNPs were enriched by oligo(dT) selection and RNA-associated proteins identified by MS. We discovered 805 proteins significantly enriched over the non-crosslinked (noCL) control (*P* < 0.01) (Fig. 1A–C; Supplemental Fig. S1A; Supplemental Table S1). As expected, Gene Ontology (GO) analysis revealed RNA-related GO terms to be significantly enriched in our data set, including “RNA metabolic process” (*P* = 8.63 × 10^−27^) and “RNA binding” (*P* = 4.58 × 10^−63^). We rediscovered 99 of 136 proteins annotated to harbor a classical RNA-binding domain (RBD) (e.g., RRM, KH; see Supplemental Table S2 for a complete list of RBDs; *P* < 0.01), highlighting the depth our RBPome and the presence of the expected molecular signatures (Fig. 1C).

### Enrichment over WCE reflects degree of poly(A)^+^ RNA association

Recent studies that compared RBPomes under different conditions have included WCE analysis to monitor whether changes in RNA binding were accompanied by changes in RBP abundance (Sysoev et al. 2016; Garcia-Moreno et al. 2019). We hypothesized that directly referencing our data to protein abundances would allow us to assess the relative RNA-binding activities of RBPs. For this, input WCEs were analyzed by MS (Fig. 1D,E; Supplemental Fig. S1B; Supplemental Table S3). Of the proteins identified by RIC, 83.7% were also detected in the WCE, which thus had a very good coverage. Background values were imputed for proteins that were not detected in the WCE (Supplemental Fig. S1C,D). WCE normalization revealed that many proteins with high MS signal in RIC were poorly captured considering their abundance. In contrast, many proteins with low to intermediate RIC signal were captured very efficiently and strongly overrepresented relative to statistical expectation based on their cellular abundance (Fig. 1E). We expect RIC/WCE ratios to reflect a combination of two RBP characteristics: (1) the fraction of the RBP that associates with poly(A)^+^ RNA in vivo, that is, its RNA-binding activity, which is determined by the affinity of the RBP for its target RNA and target RNA availability; and (2) the UV-crosslinkability of RBP and the bound RNA, which is determined by the geometry of the interaction and the type of residues present at the interface.

To validate our normalization method, we first considered proteins that we expected to have high RNA-binding activity. Through an optimal interaction surface, classical RBDs bind RNA with high affinity (Lunde et al. 2007). On average, proteins with a classical RBD were overrepresented relative to statistical expectation (Fig. 1F, left). Significant enrichment was also observed for proteins expected to have high affinity for poly(A)^+^ RNA based on GO term annotation (Fig. 1F, middle and right; Supplemental Fig. S1E).

Poor UV-crosslinkability can lead to an underestimation of the degree of RNA association for RBPs with high in vivo poly(A)^+^ RNA-binding activity. To estimate the number of false-negative data points that arise from this, we searched for known mRNA binders that failed to be enriched after RIC. Ninety-nine classical RBPs were robustly detected in RIC. In contrast, 14 were detected but not significantly enriched compared to the noCL control (*P* > 0.01) (Fig. 1C). Five never crosslinked at all, although they were present in the WCE (Fig. 1F, left, indicated by crosses). Ten of the 19 classical RBPs that were RIC-negative are known to be associated with non-poly(A) RNA species (e.g., Usp109 and Brr2/Prp28 with U1 and U5 snRNA, respectively) (Fig. 1F, left) or involved in ribosome biogenesis. Four others are meiosis-specific or mitochondrial proteins. Mitochondrially encoded transcripts are not enriched by oligo(dT) selection in *S. pombe* (Marguerat et al. 2012). Rbm8 [Y14], a component of the exon junction complex, binds spliced mRNA; but, at least in the human complex, the RNA-binding surface of its RBD is masked by Magoh and does not directly interact with RNA (Lau et al. 2003). For other RBPs, such as Vip1, the nature of the bound RNAs is unknown. However, Vip1 was identified in a screen for regulators of telomeric repeat-containing RNA (*TER1*) levels, which is non-poly(A) (Lorenzi et al. 2015).

Among proteins annotated as “mRNA binding,” 55 were robustly detected, compared to 13 that were RIC-negative. Of those, four were mitochondrial RBPs, including Mam33, homolog of a mitochondrial translation regulator in budding yeast (Fig. 1F, middle; Roloff and Henry 2015). Among proteins annotated with “mRNA metabolic process,” a higher number was inefficiently captured or did not crosslink at all (Fig. 1F, right). However, upon closer inspection, these often constituted complexes that bind deadenylated or nascent RNA lacking a poly(A) tail, for example, CCR4-NOT deadenylase and the transcription elongation factor complex (Supplemental Fig. S1F).

In summary, we observed robust enrichment of RBPs that we expected to associate with poly(A)^+^ RNA based on annotation. We take this as an indication that differences in UV-crosslinkability, although they modulate RIC/WCE ratios, do not have a dominant influence on RIC/WCE values, and that the false-negative rate for RIC is reasonably low. Because of the unknown UV-crosslinkability component, RIC/WCE ratios cannot be regarded as actual measurements of in vivo poly(A)^+^ RNA-binding activity. However, we concluded that RIC/WCE ratios can serve as an estimator of the degree of in vivo poly(A)^+^ RNA association with a certain confidence.

### Positioning the mRNA tunnel within ribosomes using RIC/WCE ratios

**Figure 2.**
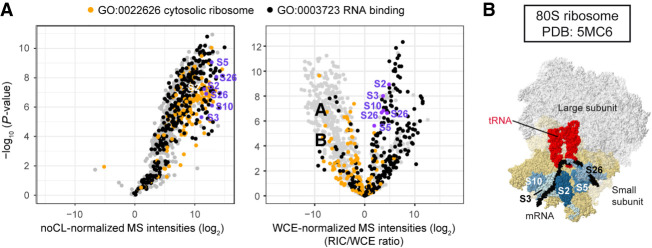
Positioning the mRNA tunnel within ribosomes using RIC/WCE-ratios. (*A*) Distribution of ribosomal proteins (RPs) in the normalized poly(A)^+^ interactome. Volcano plot of noCL-normalized (*left*) or WCE-normalized (*right*) RIC as in Figure 1D. Proteins annotated as cellular component “cytosolic ribosome” [GO:0022626] are designated in orange, with the molecular function “RNA binding” [GO:0003723] in black. For proteins annotated with both GO terms, GO:0022626 was given precedence. The RPs that were most significantly enriched on poly(A)^+^ RNA are indicated in blue. Proteins without WCE signal were disregarded (see Supplemental Figs. S1D, S2A). For reference, standard gene names for enriched RPs are given in Supplemental Fig. S2B. (*B*) Structure of the *Saccharomyces cerevisiae* 80S ribosome [PDB:5MC6] (Schmidt et al. 2016). RPs with *S. pombe* orthologs that were highly significantly enriched on poly(A)^+^ RNA are designated in blue. For reference, the mRNA is colored black, tRNAs bound in the ribosomal A- and P-sites in red, and the small and large ribosomal subunits in beige and white, respectively.

Because RIC/WCE ratios provided information about relative RNA-binding activities, we rationalized that they could be used to position RNA within large multiprotein complexes. To test this hypothesis, we analyzed enrichment of ribosomal proteins (RPs) on poly(A)^+^ RNA. In the ribosome, most structural RPs interact with rRNA, which is non-poly(A). In contrast, RPs that line the mRNA channel should be more likely to crosslink to poly(A)^+^ RNA. As predicted, most RPs were inefficiently captured. Fifteen RPs were detected in the WCEs but absent from the RIC samples, suggesting that they do not interact, even stochastically, with poly(A)^+^ RNA. However, RPs that are immediately adjacent to the mRNA channel, for example S2 and S3, were highly enriched in RIC compared to statistical expectation (Fig. 2A,B, indicated in blue). Hence, RIC/WCE ratios correlate well with expectations for protein-poly(A)^+^ RNA association based on the known structure of the ribosome (Schmidt et al. 2016). We conclude that RIC/WCE ratios allow inferences to be made about how large RNP complexes assemble on poly(A)^+^ RNA.

### RIC/WCE ratios support the proposed topology of CPF on RNA in vivo

To further validate our approach, we turned to the mRNA 3′ end processing machinery (Zhao et al. 1999; Thore and Fribourg 2019). The super-complex of CPF and CFI recognizes RNA consensus elements, but due to its complex and dynamic nature, our understanding of its interactions with pre-mRNA in vivo is limited. A structure of the *S. pombe* complex is not available, but recent studies in *S. cerevisiae* showed that CPF is organized into three modules: poly(A) polymerase, nuclease, and phosphatase (Casañal et al. 2017). Reconstitution and structural analysis of the mammalian polymerase module (CPSF160-WDR33-CPSF30-FIP1) bound to a consensus PAS element has revealed the RNA to be in direct contact with the zinc finger domains 2 and 3 of CPSF30, as well as the WD40 domain and the N-terminal region of WDR33 (Schönemann et al. 2014; Clerici et al. 2017, 2018; Sun et al. 2018). Although RNA is not present in the cryo-EM structure of the *S. cerevisiae* polymerase module, its overall organization is very similar to the mammalian module. Moreover, the residues involved in PAS recognition by CPSF30 and the WD40 domain of WDR33 are conserved in the homologous proteins Yth1p and Pfs2p, suggesting structural conservation of RNA binding between distant clades. Other components of the polymerase module, CPSF160 [Cft1p] and FIP1, are not in contact with the PAS in the structure and were proposed to act as scaffolding proteins (Clerici et al. 2018; Sun et al. 2018).

**Figure 3.**
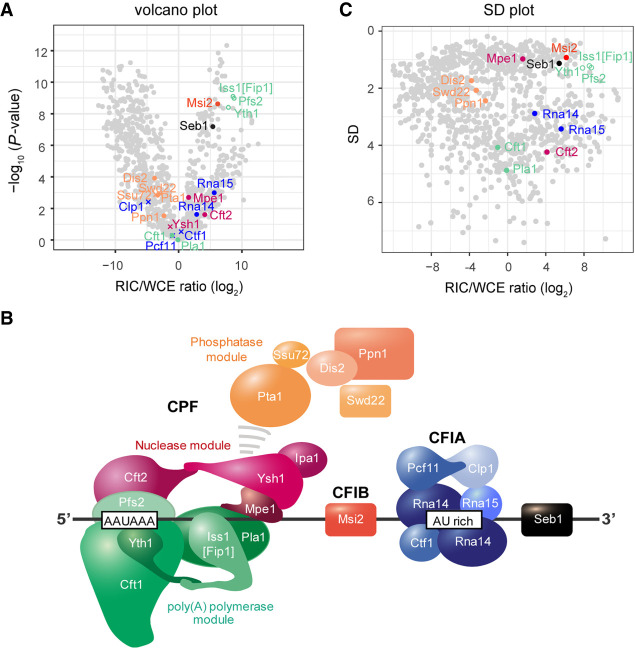
Normalized RIC data captures aspects of multiprotein complex topology. (*A*) Volcano plot of the WCE-normalized poly(A)^+^ RNA interactome as in Figure 1D. Components of CFIA, CFIB, and CPF are highlighted. (*B*) SD plot of the WCE-normalized poly(A)^+^ RNA with components of CFIA, CFIB, and CPF highlighted. SD was plotted against the fold change of mean MS intensities (log_2_) of proteins recovered from the oligo(dT) pull-downs of UV-crosslinked samples (3 J/cm^2^) over the input WCE (RIC/WCE ratios; *n* = 6). Only proteins that were detected in the oligo(dT) pull-down were included in the graph. Background values were imputed for proteins without WCE signal (empty circles). (*C*) Putative model showing the organization of the *S. pombe* 3′ end processing machinery for RNA Pol II on RNA. Overall complex organization is based on published structural data (Barnwal et al. 2012; Clerici et al. 2017, 2018; Sun et al. 2018; Hill et al. 2019).

Oligo(dT) selection during the RIC procedure limits our analysis to protein interactions with transcripts where cleavage and polyadenylation have already taken place. Nevertheless, we observed strong enrichment of several fission yeast CPF proteins on poly(A)^+^ RNA (Fig. 3A). Consistent with the described role of Pfs2p and Yth1p in PAS recognition, both factors were very strongly overrepresented in RIC. Iss1 [Fip1p] crosslinked equally well, suggesting that it could form additional contacts with RNA contributing to either stability or specificity of the CPF-RNA interaction; however, it should be noted that the three proteins were not detected in the WCE, and that enrichment values based on imputed data may be less reliable (Fig. 3A, empty circles). In contrast, other subunits of the polymerase module, Ctf1 and the poly(A) polymerase Pla1, although they crosslinked, were not strongly enriched on poly(A)^+^ RNA. This supports a role for Cft1 as a protein scaffold without strong RNA interactions, as was proposed based on the human structure. Two components of CPF's nuclease module, Mpe1 and Cft2, but not the endonuclease Ysh1, also crosslinked with poly(A)^+^ RNA, and may assist in positioning Ysh1. All subunits of the phosphatase module interacted with poly(A)^+^ RNA at low levels only. Taken together, our in vivo crosslinking data for CPF are in good agreement with the structural data from other organisms and confirm the general architecture of the complex. They support a model in which the phosphatase module either is not in direct contact with RNA, or quickly dissociates from CPF upon cleavage and polyadenylation. Moreover, RIC data suggest a more prominent role for Iss1 [Fip1p] in RNA binding than was to be expected from complexes assembled on isolated PAS elements; however, further research is needed to validate this observation.

CPF-mediated RNA cleavage is stimulated by CFI, which is recruited to auxiliary sequences at the PAS (Chen and Moore 1992). In *S. cerevisiae*, the auxiliary elements are recognized through direct interaction with the RRM domains of Rna15p and Hpr1p, which are bridged by Rna14p (Pérez-Cañadillas 2006; Pancevac et al. 2010; Barnwal et al. 2012). In our *S. pombe* RIC, Rna14, Rna15, and Msi2 [Hpr1] were significantly enriched, while other CF1A subunits—Pcf11, Clp1, and the fission yeast specific factor Ctf1—did not crosslink (Fig. 3A). The 3′ end-associated factor Seb1 was also significantly enriched on poly(A)^+^ RNA. For Seb1, RRM-dependent RNA binding and preferred recruitment to UGUAA motifs has been shown (Lemay et al. 2016; Wittmann et al. 2017). Based on our crosslinking data and the available structural information, we propose a tentative model of how the 3′ end processing machinery assembles on pre-mRNA in fission yeast (Fig. 3B).

### Concordant behavior of RNP subunits in RIC

As we analyzed CPF crosslinking, we noticed that subunits belonging to the same functional module tended to not only have similar RIC/WCE ratios but to also be alike in the statistical significance of their enrichment. For example, the polymerase module subunit Iss1 clustered with Pfs2 and Yth1, as did phosphatase module subunits (Fig. 3A). To obtain a better spread of the data, we plotted RIC/WCE ratios against their standard deviation. Consistent with the previous observation, components of the same modules clustered in similar regions of the plot (Fig. 3C). In short, different modules of the complex appeared to not only be defined by their degree of association with poly(A)^+^ RNA (RIC/WCE ratio), but to also be characterized by a submodule-typical noisiness of the data (SD). To our understanding, this “conservation of noisiness” is an intrinsic property of the RIC method. We can think of two possible explanations for this behavior: First, it appears that MS intensities of proteins that are stably associated show higher correlation between replicates than with other proteins. We consider it plausible that this reflects biological (expression level) noise or technical variability in extract preparation (e.g., varying solubilization of chromatin between samples), which would tend to affect members of the same complexes similarly. Second, there are systematic aspects to the technical noise during MS data acquisition, which are primarily connected to signal strength. Because different subunits of stable RNA-binding complexes are likely to have comparable signals both in the WCE and in RIC, the noise of RIC/WCE ratios could be influenced by the stoichiometries of RNA–protein interactions through this as well. We infer that clustering in the SD plot is more typically observed for modules that are preferentially present on RNA as fully assembled complexes, rather than as individual components that bind in a noncorrelated fashion. We conclude that RIC-based analysis can provide insights into the organization of native RNA-regulatory complexes and help to generate functional models that can be experimentally tested in the future.

### RNA exosome regulatory factors are RNA-binding proteins

Next, we examined RNA interactions of the nuclear 3′-5′ RNA decay machinery. The RNA exosome regulates levels of various cellular RNAs (Kilchert et al. 2016; Morton et al. 2018). On its own, the exosome is an unspecific RNA-degrading enzyme with low intrinsic nuclease activity; it requires cofactors to ensure selective and efficient degradation of its substrates (Schmid and Jensen 2019; Kilchert 2020). Conserved Ski2-like RNA helicases are thought to constitute targeting platforms for various substrate-selecting cofactors. They associate with the top of the exosome and facilitate RNP disassembly and RNA threading into the active complex (Weick et al. 2018). Fission yeast has two essential nuclear Ski2-like helicases, Mtr4 and Mtl1 (Mtr4-like 1), which localize to the nucleolus and the nucleoplasm, respectively, and mediate degradation of different substrates (Bühler et al. 2007; Lee et al. 2013; Egan et al. 2014; Zhou et al. 2015).

Mtl1 and Mtr4 have been found to interact with multiple proteins in affinity pull-downs of endogenous complexes, revealing similarities between the exosome regulatory networks of fission yeast and higher eukaryotes, but not *S. cerevisiae* (Supplemental Fig. S3A; Lee et al. 2013; Egan et al. 2014; Zhou et al. 2015; Tudek et al. 2018; Silla et al. 2020): *S. pombe* Mtl1 and the zinc finger protein Red1 form a core complex (MTREC) associated with the nuclear poly(A)-binding protein Pab2, Red5 [Z3CH3] and Rmn1 [RBM26/27] that strongly resembles human Poly(A) Tail eXosome Targeting (PAXT), which targets processed transcripts (Meola et al. 2016; Silla et al. 2020). Like human MTR4, Mtl1 connects to the nuclear cap-binding complex CBC-Ars (Cbc1-Cbc2-Pir2 [ARS2]) that has widespread functions in nuclear mRNA metabolism (Andersen et al. 2013; Meola et al. 2016). In addition, Mtl1 interacts with Mmi1, a YTH domain protein that recognizes TNAAAC motifs on RNA and induces exosome-dependent degradation in complex with Iss10 (Harigaya et al. 2006; Yamashita et al. 2012; Shah et al. 2014; Kilchert et al. 2015).

According to our RIC data, most factors linked to the exosome by previous studies, including Red1, Mtl1, Mmi1, Iss10, Cbc1, Cbc2, Pir2 [ARS2], Pab2, and Red5 (but not Erh1, Rmn1, Nrl1, Ctr1) were strongly enriched on poly(A)^+^ RNA (Supplemental Fig. S3B), supporting a potential contribution of these proteins to different aspects of exosome targeting to substrate RNAs. Consistent with the current model for the exosome regulatory network in fission yeast, clustering in the SD plot was observed for CBC-Ars2 and MTREC, and to a lesser degree for other modules (Supplemental Fig. S3A,B).

### Comparative RIC with exosome mutants reveals differences in substrate accessibility

**Figure 4.**
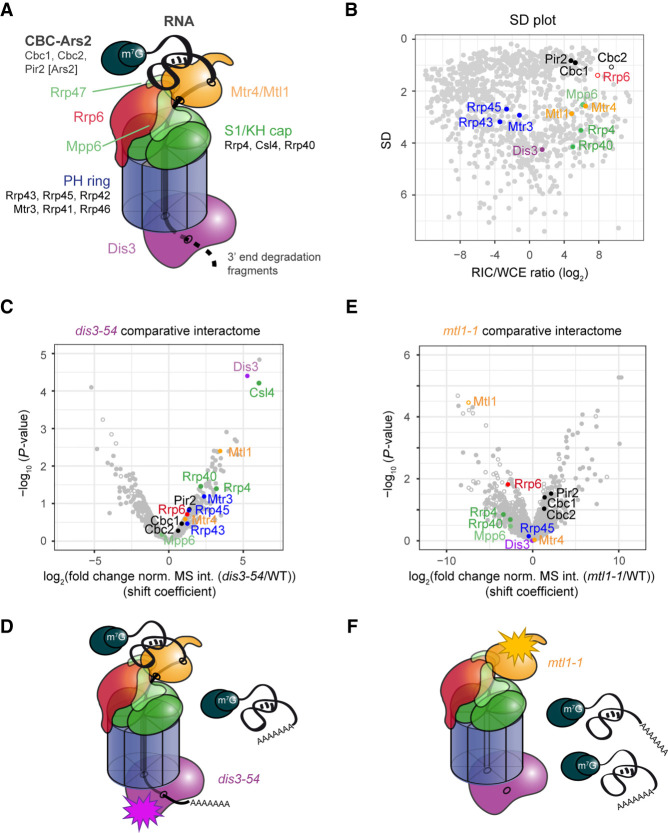
Comparative RIC with exosome mutants captures quantitative differences in RNA channeling. (*A*) Schematics of the nuclear RNA exosome complex based on crystal structures of the complex (Makino et al. 2013, 2015; Schuch et al. 2014; Zinder et al. 2016). (*B*) SD plot of the WCE-normalized poly(A)^+^ RNA interactome as in Figure 3B with components of nuclear exosome highlighted. (*C*) Volcano plot of a comparative RIC experiment for *dis3-54*. *P*-values (−log, moderated Student's *t*-test) for the comparison between RIC/WCE ratios of mutant and WT interactomes were plotted against the fold change of RIC/WCE ratios between mutant and WT (shift coefficient; *n* = 3). Components of the nuclear exosome are highlighted. Full circles denote proteins that were detected in the mutant interactome, and empty circles denote proteins that were not detected (but were present in WT). Shift coefficients for individual proteins can be found in Supplemental Table S4. (*D*) Impairment of Dis3 exonucleolytic activity leads to accumulation of nuclear RNPs, a part of which associate with the exosome complex but fail to be degraded. (*E*) Volcano plot of a comparative RIC experiment for *mtl1-1* as in *C*. (*F*) Mtl1 facilitates engagement of the exosome complex with substrate RNA. In the mutant, nuclear RNPs accumulate but do not engage with the exosome complex.

The structure of the exosome complex is well described: The core components form a catalytically inert barrel through which RNA is threaded before being degraded by Dis3 exonuclease, which is situated at the bottom of the structure (Fig. 4A; Makino et al. 2013, 2015; Schuch et al. 2014; Zinder et al. 2016). Alternatively, RNA can be degraded by Rrp6 exonuclease at the top of the complex. In addition, variant structures suggest that RNA can access Dis3 directly without passing through the channel (“direct access,” *da*) (Makino et al. 2013; Liu et al. 2014). In vivo data from budding yeast have identified short structured RNAs as primary *da* substrates, but it is currently unclear whether exosome complexes in other eukaryotes also adopt the *da* conformation (Han and van Hoof 2016; Delan-Forino et al. 2017; Gerlach et al. 2018; Weick et al. 2018)

In agreement with the structural data, RIC analysis revealed that components of the cap region are enriched on poly(A)^+^, which supports its proposed function in RNA selection (Fig. 4B; Supplemental Fig. S3C, in green/yellow). In contrast, proteins that constitute the PH ring of the core showed limited interactions with poly(A)^+^ RNA (Fig. 4B; Supplemental Fig. S3C, in blue). We assume this to be the case because removal of the poly(A) tail during degradation prevents capture of the crosslinked complex by oligo(dT) capture. In accordance with this assumption, recovery of crosslinked exosome core subunits from HEK293 cells was reported to be more efficient when crosslinked RNPs were enriched by chemical extraction rather than poly(A)^+^ RNA selection (Urdaneta et al. 2019). As observed for other stable complexes, components of the ring and cap showed distinctive clustering in the SD plot (Fig. 4B).

The channel-threading conformation is thought to be the preferred state of the nuclear exosome (Bonneau et al. 2009; Schneider et al. 2012; Delan-Forino et al. 2017). When we performed RIC with *dis3-54*, a mutant with reduced exonucleolytic activity (Murakami et al. 2007), crosslinking to poly(A)^+^ RNA was strongly increased all along the exosome barrel and cap (Fig. 4C). We hypothesize that the inability of the *dis3* mutant to efficiently degrade RNA leads to continuous association of poly(A)^+^ RNA with the channel, underlining the prominent role for the channel in RNA decay (Fig. 4D).

To assess the contribution of Mtl1 to substrate channeling, we used an *mtl1-1* mutant that carries six point mutations near the arch domain (Supplemental Fig. S3D; Lee et al. 2013). Levels of the mutant protein were comparable to WT (Supplemental Fig. S3E). Crosslinking of Mtl1 to poly(A)^+^ RNA was lost in the mutated protein (Fig. 4E), suggesting that the arch domain might contribute to RNA binding. In addition, association of poly(A)^+^ RNA with the cap region was strongly reduced (negative shift in the comparative volcano plot) (Fig. 4E; Supplemental Table S4). Concomitantly, nuclear RNPs marked by CBC-Ars2 were stabilized (positive shift). RNA association of the other exosome-associated helicase Mtr4 was unchanged. This suggests that a significant proportion of nuclear poly(A)^+^ RNA substrates depends on Mtl1 for recruitment to the exosome and supports a model in which channel-dependent RNA degradation is facilitated by the presence of functional Mtl1 (Fig. 4F). All cap-associated proteins shifted by a similar amount when comparing WT to mutant. Similarly, CBC-Ars2 shifted as a cluster, suggesting that we detect protein–RNA interactions of the assembled complex.

**Figure 5.**
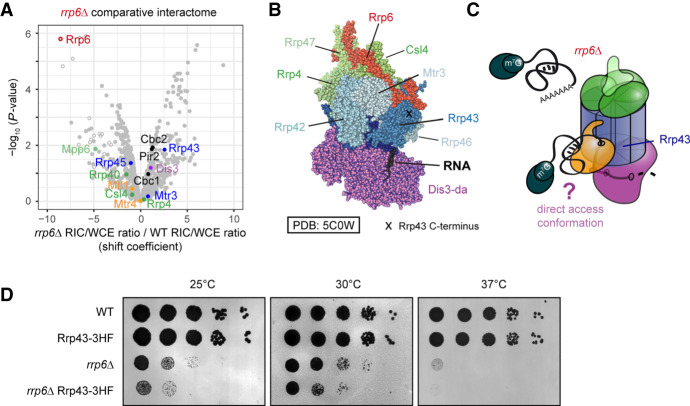
Altered substrate access to the exosome complex in *rrp6Δ*. (*A*) Some exosome subunits show increased association with poly(A)^+^ RNA in *rrp6Δ*. Volcano plot of a comparative RIC experiment for *rrp6Δ* as in Figure 4C. (*B*) Structure of the *S. cerevisiae* nuclear exosome complex in the direct access conformation (PDB: 5C0W) (Makino et al. 2015). Positions of various subunits and of the C-terminus of Rrp43 are marked. (*C*) In the absence of Rrp6, nuclear RNPs accumulate but fail to engage with the exosome cap region. At the same time, increased RNA crosslinking with Dis3 and Rrp43 could suggest a potential rerouting of poly(A)^+^ RNA substrates from the channeling route to direct access. (*D*) In a plate-based growth assay, addition of a C-terminal tag to Rrp43 results in a slow growth phenotype when combined with *rrp6Δ*, but not in a wild-type background. Serial dilutions (1:10) of the indicated yeast strains were spotted on YES and incubated at the indicated temperatures for several days.

Similarly, deletion of *rrp6* reduced RNA recruitment to the cap region and to Mtl1 helicase (Fig. 5A). In *S. cerevisiae* and humans, Rrp6p/EXOSC10 was shown to play a structural role in anchoring Mtr4 to the exosome that can involve a conformational change (Schuch et al. 2014; Makino et al. 2015; Gerlach et al. 2018; Weick et al. 2018), raising the possibility that loss of RNA recruitment to the exosome in *rrp6Δ* is a consequence of compromised anchoring of Mtl1. As in *mtl1-1*, we observed a concomitant stabilization of nuclear RNPs, supporting a role for Rrp6 in promoting channel threading. In addition, we detected a novel signature in *rrp6Δ* comparative RIC: a coshift of Dis3 and Rrp43. Both proteins crosslinked better to poly(A)^+^ RNA than in WT (Fig. 5A). In *S. cerevisiae*, Dis3p rotates to adopt the *da* conformation that allows RNA substrates to directly enter the exonucleolytic center, bypassing the channel (Bonneau et al. 2009; Makino et al. 2013, 2015; Liu et al. 2014). In this conformation, substrate RNA passes Rrp43p on the outside of the PH ring, which may favor crosslinking of Rrp43p to *da* substrates (Fig. 5B). Hence, the coshift of Dis3 and Rrp43 in comparative RIC could reflect increased use of the *da* route by poly(A)^+^ RNA in *rrp6Δ* (Fig. 5C). We have at present no direct evidence that the *da* conformation does indeed occur in *S. pombe*, and further research is needed to validate the hypothesis. However, an *S. cerevisiae* mutant that is unable to adopt the *da* conformation, and that has no discernible growth phenotype, shows a synthetic growth defect with *rrp6Δ* (Han and van Hoof 2016), documenting increased physiological importance of *da* in the absence of Rrp6p in budding yeast. Likewise, when we added a C-terminal tag to *S. pombe* Rrp43, we observed no growth impairment in WT, but synthetic slow growth when tagged Rrp43 was combined with *rrp6Δ* (Fig. 5D), possibly because addition of a protein tag at this position interferes with substrate recruitment/access to the *da* route. If true, adaptation of the *da* conformation could contribute to the functional redundancy between Dis3 and Rrp6 that allows efficient regulation of cellular RNA levels.

### Classification of nonclassical RBDs based on RIC/WCE ratios

**Figure 6.**
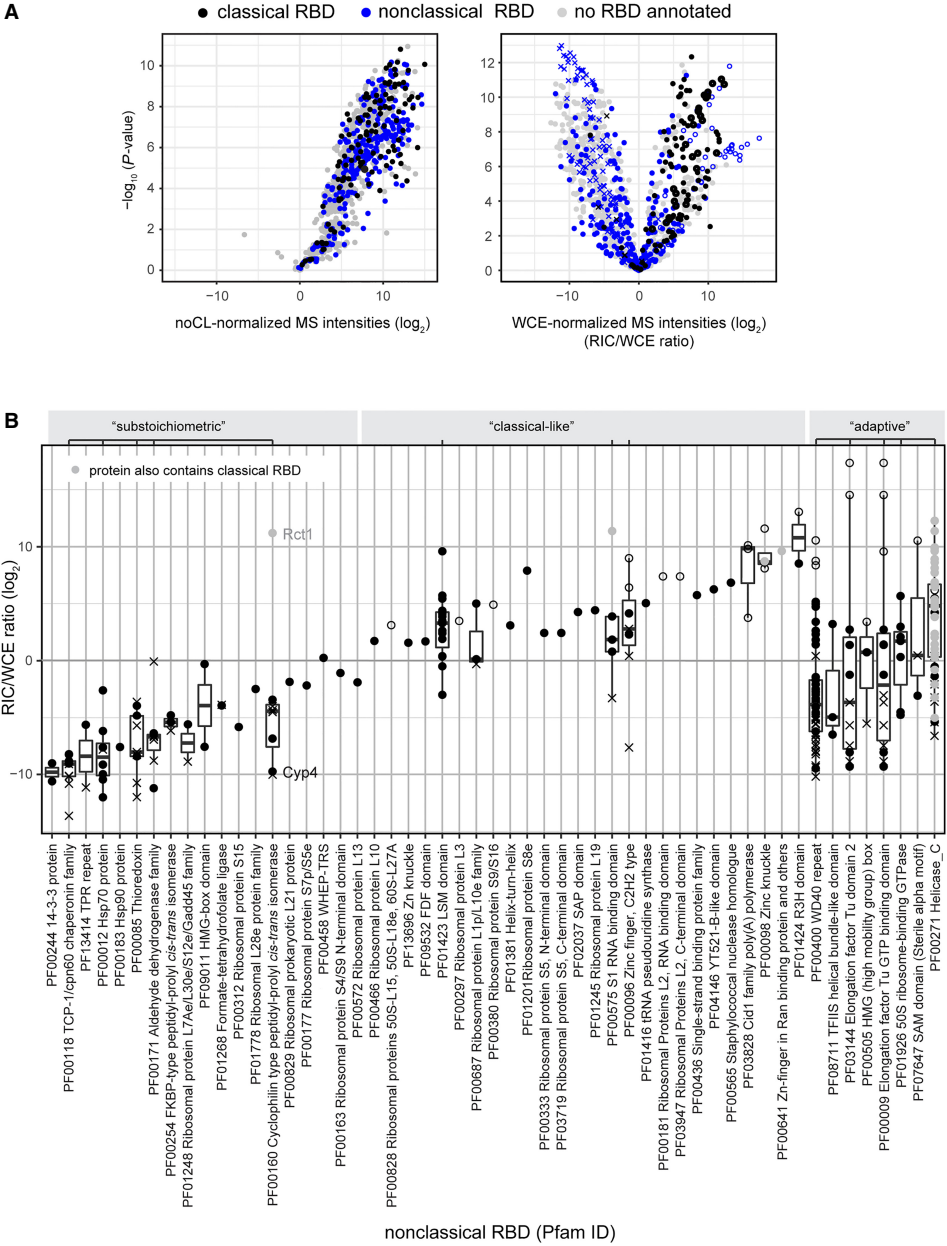
Properties of nonclassical RBDs. (*A*) Distribution of proteins annotated with a classical or nonclassical RBD in the noCL-normalized (*left*) and WCE-normalized poly(A)^+^ RNA interactome (*right*) as in Figure 1C. Nonclassical RBDs comprise 192 Pfam identifiers derived from the initial human interactome as well as any domain in RBDmap that had at least three peptides supporting it (Castello et al. 2012, 2016; Moore et al. 2018). The complete list of Pfam identifiers used for this analysis can be found in Supplemental Table S2. (*B*) Boxplot of RIC/WCE ratios for all proteins annotated with a nonclassical RBD that were detected in WT poly(A)^+^ RIC using symbols to indicate protein populations as in Figure 1D. Proteins that also harbor a classical RBD are marked in gray. Proteins annotated with GO:0022626 [cellular component: cytosolic ribosome] are not included. Abundant nonclassical domains (where at least four different domain-containing proteins were detected in the experiment) were classified as either “substoichiometric,” “classical-like,” or “adaptive,” as indicated *above* the plot.

Based on the RIC data, we also sought to characterize RBPs that lack classical RBDs. For many novel RBPs, the mode of RNA binding is not well established. A recent method, RBDmap, has adapted the RIC workflow to identify the regions of RBPs engaged in RNA binding (Castello et al. 2016). Together, annotated noncanonical RBDs and RNA-binding regions discovered by RBDmap comprise 192 Pfam identifiers (Supplemental Table S2; Castello et al. 2012, 2016; Moore et al. 2018), and of these, 148 occur in *S. pombe*. Overall, the set of proteins recovered in *S. pombe* RIC agrees very well with studies from other organisms that have greatly expanded the scope of known RBPs (Fig. 6A, left; Baltz et al. 2012; Castello et al. 2012, 2016; Kwon et al. 2013; Mitchell et al. 2013; Beckmann et al. 2015; Matia-González et al. 2015). When we calculated RIC/WCE ratios, the behavior of nonclassical RBPs differed markedly from classical RBPs: Many nonclassical RBPs have low RIC/WCE ratios, indicating that they interact with RNA but are strongly underrepresented in RIC compared to other RBPs (Fig. 6A, right). We conclude that in stark contrast to classical RBPs, many RBPs with nonclassical RBDs that were defined based on UV-crosslinking experiments have weak RNA-binding activity in vivo. We expect these to associate with poly(A)^+^ RNA at substoichiometric levels under standard growth conditions. However, nonclassical RBPs display a broad range of RIC/WCE ratios, from low to very high (Fig. 6B). For many nonclassical RBDs the number of annotated proteins in our data set is too low to allow reliable assignment of average RNA-binding activities. Among RBDs present in at least four different proteins detected in our RIC experiment, we can distinguish (1) domains found in proteins with high RIC/WCE ratios, which we refer to as “classical-like” (including LSM, S1, and C2H2-type zinc finger domains) and are likely to represent professional RBDs that associate with RNA almost constantly; (2) domains found in proteins with low RIC/WCE ratio, that we refer to as “substoichiometric” (including TCP-1/cpn60 chaperonin family proteins, Hsp70 proteins, thioredoxins, aldehyde dehydrogenase family proteins, and cyclophilin-type peptidyl-prolyl *cis-trans* isomerases); and (3) domains found in proteins with a broad range of RIC/WCE ratios, which we refer to as “adaptive” RBDs (including WD40 repeat proteins, Elongation factor Tu domain 2-containing proteins, Elongation factor Tu GTPase domain, 50S ribosome-binding GTPase, and Helicase C domain-containing proteins). In the case of WD40 repeats, a common protein–protein interaction fold, the presence of basic amino acids in the binding surface was previously found to correlate with its ability to bind RNA (Castello et al. 2012, 2016).

### A subpopulation of the substoichiometric RBP Cyp4 interacts with RNA

**Figure 7.**
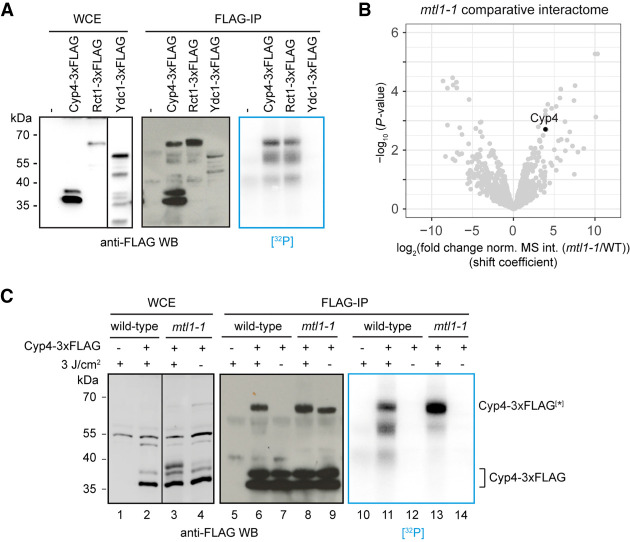
Only a subpopulation of the substoichiometric RBP Cyp4 interacts with RNA. (*A*) Crosslinking and immunoprecipitation (CLIP) analysis of FLAG-tagged proteins captured from WCEs of 4sU-labeled UV-crosslinked cells (3J/cm^2^). After RNase digest, 5′ ends of crosslinked RNAs were radioactively labeled using T4 polynucleotide kinase and [γ-^32^P]ATP, and complexes were separated by gel electrophoresis followed by membrane transfer. A nontagged strain was included as control. All shown WCE samples are from the same membrane at the same exposure; irrelevant lanes were removed. (*B*) Volcano plot of a comparative RIC experiment for *mtl1-1* as in 4E. Poly(A)^+^ RNA association of Cyp4 is increased compared to WT. (*C*) CLIP analysis of FLAG-tagged Cyp4 in wild-type or *mtl1-1* as in *A*. Non-irradiated cells and a nontagged strain were included as controls. (*) Cyp4 species that migrates at a higher apparent molecular weight. Although the band is apparently stabilized by crosslinking, it is present in noCL samples where no associated RNA can be detected (lanes *9* and *14*). All shown WCE samples are from the same membrane at the same exposure; irrelevant lanes were removed.

Because substoichiometric RBPs were strongly underrepresented in RIC compared to statistical expectation, we decided to validate RNA binding of a representative example experimentally. Cyp4 is a cyclophilin B–type peptidyl-prolyl isomerase, a class of ER-resident enzymes with a nuclear subpopulation (Dieriks and Van Oostveldt 2012). Its human ortholog mediates retro-translocation of peptide hormones from the secretory pathway to the nucleus, where they act as transcription factors (Rycyzyn et al. 2000; Rycyzyn and Clevenger 2002). To assay RNA binding of Cyp4, we performed CLIP followed by 5′ labeling of crosslinked RNA with ^32^P (Fig. 7A). An RRM-containing cyclophilin with strong enrichment in RIC, Rct1, served as positive control. Radioactive labeling was observed for the control RBP, as expected. In contrast, there was no detectable signal for the predominant Cyp4 species (apparent molecular weight [MW] 35–40 kDa). However, a subpopulation of Cyp4 that migrated higher was enriched during IP and crosslinked very efficiently (Fig. 7A; Supplemental Fig. S4). Cyp4 is one of several proteins that are more enriched on poly(A)^+^ RNA in the *mtl1-1* mutant (Fig. 7B). This coincides with elevated levels of the higher apparent MW species in the mutant WCE and increased signal in CLIP (Fig. 7C). At present, the nature of the higher MW species remains unknown. Potential causes for the size shift include post-translational modification, formation of an SDS-stable dimer, or a variant protein isoform, but more research is needed to clarify. Overall, the CLIP data support that Cyp4 is a substoichiometric RBP as predicted by RIC. Moreover, they suggest that the cause for substoichiometric binding, rather than Cyp4 having low overall affinity for RNA, could be that RNA binding is limited to a molecularly defined subpopulation of the protein that is present at low levels under standard growth conditions.

### High RNA-binding activity is frequent among transcription-related proteins

**Figure 8.**
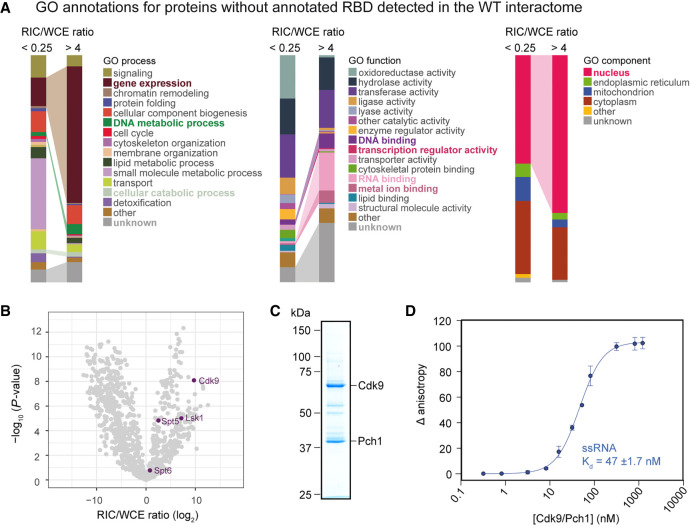
Characterization of RBPs with high RNA-binding activity that lack annotated RBDs. (*A*) Comparison of GO term distribution for RNA interactors without annotated RBDs that are either underrepresented (RIC/WCE ratio <0.25; *n* = 304) or enriched (RIC/WCE ratio >4; *n* = 203) in poly(A)^+^ RIC compared to statistical expectation (*P* < 0.01), visualized with the QuiLT tool on PomBase (Lock et al. 2019). Categories overrepresented among proteins enriched in RIC are highlighted in bold. (*B*) Volcano plot of the WCE-normalized RIC as in 1C. The cyclin-dependent kinases Cdk9 and Lsk1 and the transcription elongation factors Spt5 and Spt6 are enriched on poly(A)^+^ RNA. (*C*) Coomassie stain of Cdk9/Pch1 purified from insect cells. (*D*) Fluorescence anisotropy assay measuring binding of increasing amounts of purified Cdk9/Pch1 to 8 nM FAM-labeled RNA.

Although RIC/WCE ratios of many nonclassical RBPs were low, 202 proteins that were not annotated with any classical or nonclassical RBD from our list had high in vivo poly(A)^+^ RNA-binding activity according to our analysis (RIC/WCE ratio >4, *P* < 0.01) (Supplemental Table S5). When comparing these to RBPs that were underrepresented (RIC/WCE ratio <0.25, *P* < 0.01), enriched proteins were more likely to be nuclear; to be involved in gene expression and DNA metabolic processes; to be annotated as RNA-, DNA-, or metal-ion-binding; or to possess transcription regulator activity (Fig. 8A). We were struck by the high representation of DNA- or transcription-related GO terms among highly active RNA binders. Recently, RIC of human nuclei identified multiple proteins with dual specificity for DNA/RNA, and an increasing number of studies have reported cases of chromatin-associated proteins that are bound and/or regulated by RNA binding (Conrad et al. 2016; Hendrickson et al. 2016). A prominent example is the PRC2 complex, a key chromatin modifier that binds RNA promiscuously via noncanonical RNA-binding elements that are dispersed across the surface of the complex (Davidovich et al. 2013; Cifuentes-Rojas et al. 2014; Kaneko et al. 2014; Long et al. 2017). In *S. pombe* RIC, the following proteins with GO term “histone modification” were significantly enriched on poly(A)^+^ RNA: the histone deacetylase complex subunit Sap18; the argonaute protein Ago1; and the rRNA methyltransferase and potential histone H2A methyltransferase Fib1; and Spac5g10.01, a homolog of TRRAP, which is a component of the histone acetylation complex in humans. In addition, a number of DNA-binding transcription factors was highly enriched on poly(A)^+^ RNA, including the shuttle craft/ NFX1 homolog Spcc18.03, and a calcineurin-responsive transcription factor, Prz1 (Supplemental Fig. S5A).

Among all chromatin-associated RBPs without annotated RBD, Cdk9 was most highly enriched compared to statistical expectation (Fig. 8B; Supplemental Table S5). Cdk9 (P-TEFb) is a cyclin-dependent protein kinase that controls early elongation of RNA Pol II (Price 2000). Although its function is protein-directed, it has previously been observed to associate with RNA (Brannan et al. 2016; Battaglia et al. 2017; Trendel et al. 2019). When bound to the cyclin Pch1, Cdk9 phosphorylates various regulators of transcription elongation, including Spt5 (Pei et al. 2003). To note, Spt5 is also highly enriched in RIC, as is a paralog of Cdk9, Lsk1 (Fig. 8B). In *S. cerevisiae*, the orthologous cyclin-dependent Pol II kinase complex Ctk1/Ctk2/Ctk3 (Lsk1/Lsc1 in *S. pombe*) was recently shown to bind RNA in the nanomolar range (Battaglia et al. 2017). To assess RNA binding of *S. pombe* Cdk9 in vitro, we coexpressed full-length *S. pombe* Cdk9 together with its cognate cyclin Pch1 (Fig. 8C); functionality of the purified complex was validated by in vitro kinase assay (Supplemental Fig. S5C,D). RNA binding of Cdk9/Pch1 was then assessed by fluorescence anisotropy assay (Fig. 8D). Cdk9/Pch1 bound a generic FAM-labeled RNA oligo with high affinity (*K*_*d*_ = 47 ± 1.7 nM), confirming that our normalization approach successfully identifies RBPs that are characterized by high RNA-binding activity.

## Discussion

**Figure 9.**
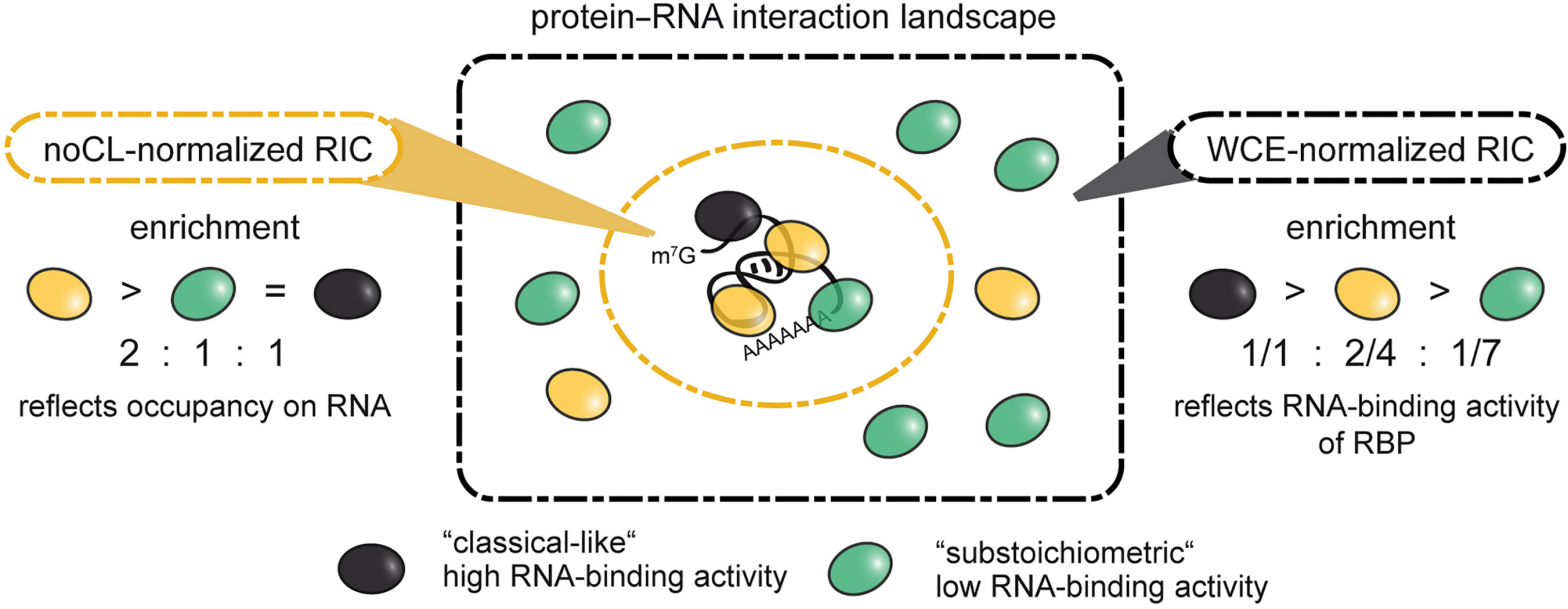
Depending on the mode of normalization, RIC captures different aspects of the protein–RNA interaction landscape. Normalization to a non-crosslinked control yields a quantitative snapshot of the partial proteome that an average RNA molecule interacts with (*left*). Here, enrichment values represent occupancy of a given RBP on RNA. In contrast, normalization to protein abundances will yield the relative enrichment of RBPs in the interactome compared to statistical expectation, thus providing an estimate of the fraction of an RBP that is associated with poly(A)^+^ RNA (*right*). Here, enrichment values represent in vivo poly(A)^+^ RNA-binding activity of a given RBP. The numbers represent an idealized case for the purpose of illustration.

To assess relative RNA-binding activities of fission yeast RBPs proteome-wide, we have combined poly(A)^+^ RIC with WCE normalization to control for protein abundances. With this approach, we were able to complement the “snapshot” of the RBPome, which assesses occupancy of proteins on RNA, with robust and quantitative information on the degree of protein–RNA association in vivo (Fig. 9).

RIC and related techniques have identified a multitude of proteins capable of interacting with RNA in different cell types of various organisms (Baltz et al. 2012; Castello et al. 2012, 2016; Kwon et al. 2013; Mitchell et al. 2013; Beckmann et al. 2015; Matia-González et al. 2015; Conrad et al. 2016; Sysoev et al. 2016; Marondedze et al. 2019). Overall, the same classes of proteins were identified over and over again, and RNA–protein interactions appear to be remarkably conserved. However, with the identification of novel RBPs—and the recurrent detection in RNA interactomes of proteins with well-described primary functions that are not RNA-related, such as metabolic enzymes—came the debate of how prevalent the observed interactions are in the cell. Here, we have used *S. pombe* RNA-binding activity estimates to classify novel RBDs that were previously identified in human capture experiments (Castello et al. 2012, 2016). Several of these domains were consistently found in proteins that were highly enriched on poly(A)^+^ RNA and behaved just like classical RBPs. Many others reproducibly crosslinked, but were very much underrepresented in the RNA interactome compared to statistical expectation. These substoichiometric RBPs with low RIC/WCE ratios are clearly an intriguing class. Because of the limitations of the oligo(dT) selection procedure, some of these RBPs may preferentially bind non-poly(A) RNA species. Likely examples are Nhp2 and Snu13, which are components of box H/ACA and U3 snoRNPs, respectively. Others, however, may either bind to RNA with low affinity or display “moonlighting” RNA-binding activities (Cieśla 2006; Castello et al. 2015). For example, mouse glyceraldehyde-3-phosphate dehydrogenase (GAPDH), a glycolytic enzyme that is also present in the *S. pombe* RNA interactome, was previously reported to bind and translationally inhibit IFNG-mRNA in a manner that is inversely correlated with the glycolytic activity of the cell, and only a fraction of GAPDH molecules associates with RNA during normal growth (Chang et al. 2013). Human aconitase 1 (ACO1), also known as iron-responsive protein 1 (IRP1), becomes an RBP when it loses its iron-sulfur cluster at low iron levels (Muckenthaler et al. 2017); according to our RIC data, *S. pombe* Aco1 is a substoichiometric RBP. Similarly, RNA binding of proline *cis-trans* isomerases and chaperones is increased in HEK293 cells upon virus infection (Garcia-Moreno et al. 2019). We observe the same for *S. pombe* Cyp4 in the *mtl1-1* mutant, although the underlying biology remains unclear at present. In short, many proteins from the growing list of enzymes confirmed to moonlight as RNA binders and/or be regulated through an RNA-binding event are among the substoichiometric RBPs that we define based on RIC/WCE ratios, underlining that RNA binding is a secondary function for many of these proteins. In support of this, 3.6% of proteins that contain a nonclassical RBD and bind RNA in the substoichiometric range are annotated with the GO term “mRNA metabolic process,” which is close to the proteome-wide average of 6.2%. In contrast, 13.1% of proteins with RBDs that we classified as “adaptive” are annotated with the term, and 30% of those that we defined as “classical-like” (Fig. 2B).

We expect RNA-binding characteristics of RBDs to be highly conserved. Owing to the low number of annotations in *S. pombe*, the assignment of nonclassical RBDs to the different classes would greatly benefit from the inclusion of data from other species. In addition, comparison to alternative crosslinking methods, such as formaldehyde crosslinking, could help to reduce crosslinking bias. Likewise, the data should be complemented by non-poly(A) RIC data, for which several methods have recently been developed (Asencio et al. 2018; Shchepachev et al. 2019; Trendel et al. 2019; Urdaneta et al. 2019); this would help to clean up the substoichiometric category of non-poly(A) RNA binders and pave the way for a comprehensive classification of RBDs based on in vivo RNA-binding activity. As another caveat, RIC measures enrichment of proteins, not protein domains. For multidomain RBPs, this can lead to an overestimation of the RNA-binding activity for individual RBDs. To exclude multidomain effects, WCE normalization could be combined with the RBDmap approach (Castello et al. 2016). Although all these limitations apply to the set of substoichiometric RBDs we present here, we consider the attempt worthwhile and a refined classification highly desirable: By predicting whether RNA is the primary bound substrate or more likely to be a secondary target, it facilitates the interpretation of domain annotations in uncharacterized proteins and can help to pinpoint regulated binding events.

WCE-normalized RIC is one approach that gives a quantitative measure of the relative RNA-binding activities of different RBPs. Recently, orthogonal approaches have been developed to study RNA–protein interactions, for example R-DeeP, a density gradient centrifugation method that compares sedimentation of protein complexes before and after RNase treatment (Caudron-Herger et al. 2019). Here, the fraction of an RBP that co-sediments with RNA reflects its RNA-binding activity. Because samples are not crosslinked, we expect R-DeeP to perform particularly well for stable RNA–protein interactions, and maybe less well for RBPs with rapid on-off kinetics. By design, R-DeeP treats complexes as one unit and reflects RNA-binding activities of full RNPs rather than of individual subunits. In this respect, it differs from RIC, and the information gained from both methods is complementary. Alternatively, the strength of PAR-CLIP signal above background has been used to compare RBPs, for example, a set of 14 transcription elongation factors from *S. cerevisiae* (Battaglia et al. 2017). In contrast to RIC, PAR-CLIP is limited to a relatively low number of samples at a time, the true power of the method being that it maps RNA–protein interactions at nucleotide resolution. Although Battaglia et al. (2017) report a predominant association with uncleaved pre-mRNA and our RIC data set is limited to processed transcripts, the resulting RNA-binding activity estimates of both methods are in good agreement: Of the factors with high PAR-CLIP signal in *S. cerevisiae*—Spt5p, Set1p, Ctk1p [Lsk1], Spt6p, Ctk2p [Lsc1], and Bur1p [Cdk9]—three (Spt5, Lsk1, and Cdk9) were highly enriched in *S. pombe* RIC, one moderately (Spt6). The cyclin Lsc1 and the histone modifier Set1 were not detected. Because all of these factors were described to bind nascent pre-mRNAs, the strong enrichment of Spt5, Cdk9, and Lsk1 on poly(A)^+^ RNA was unexpected. It suggests that these TEFs stay associated with mRNAs post-transcriptionally and might interact with RNA independently of the Pol II transcription machinery. In contrast, we did not detect poly(A)^+^ RNA association for most components of the PAF complex, nor for the histone modifier Set1, suggesting that their interactions with RNA may be limited to nascent transcripts. These potential differences in dissociation kinetics should be validated in future experiments.

Among annotated serine/threonine kinases, Cdk9 and Lsk1 were particularly enriched on poly(A)^+^ RNA. Comparable poly(A)^+^ RNA-binding activities were only observed for the atypical protein kinases Rio1/2 (Supplemental Fig. S5B). Rio kinases have a conserved role in rRNA maturation and were recently reported to be regulated by binding to rRNA (Knüppel et al. 2018). However, a broader role for Rio kinases in regulating nutrient-activated expression of mRNA genes has been proposed (Iacovella et al. 2018). Our data support a model in which Rio kinase activity could be regulated by more diverse RNA species.

At the outset, we were particularly interested in adapting RIC as a tool to study RNP complex topology in vivo. Using the 3′ end processing machinery as an example, we show that RIC/WCE ratios allow subunits that are directly involved in RNA binding within large RNA–protein assemblies to be pinpointed. In addition, we show that dynamic changes in the RNA-binding behavior of large RNPs can be captured by RIC. Specific RNP conformations will generally be defined by a set of conformation-specific RNA–protein interactions. Although RIC/WCE ratios derived from single-condition RIC represent average values for the ensemble of RNP states and thus do not allow easy identification of conformation-specific RNA–protein interaction signatures, one can delimit discrete topological states of dynamic RNPs by comparing between conditions and identifying covariant patterns. We apply this strategy to monitor changes in substrate access to the RNA exosome in exosome mutants. We propose that this approach can be used to identify RNP remodeling events under various conditions and thus provide insights into the function of multi-subunit RNA-regulatory complexes.

## Methods

### Yeast strains and antibodies

*S. pombe* strains used in this study are listed in Supplemental Table S6. Standard methods were used for cell growth and genetic manipulations (Moreno et al. 1991). Cells were grown in yeast extract with supplements (YES) at 30°C unless indicated otherwise. The following antibodies were used: Peroxidase Anti-Peroxidase Soluble Complex antibody (Sigma-Aldrich P1291), anti-FLAG MS2 (Sigma-Aldrich F3165), anti-GAPDH/Gpd3, clone GA1R (Biomol MM-0163-P).

### Poly(A)^+^ RNA interactome capture (RIC)

Poly(A)^+^ RNA interactome capture in *S. pombe* was essentially performed as described for *S. cerevisiae* (Beckmann et al. 2015), with minor modifications: Cells were grown in 1 L EMMG with limited amounts of uracil (10 mg/L) and labeled with 4sU (1 mg/L) for 4.5 h, then harvested by filtration, UV-crosslinked at 365 nm at 3J/cm^2^ in 50 mL PBS, and snap-frozen in liquid N_2_. Cells from 3 × 1 L of culture were pooled per sample and lysed by grinding in liquid N_2_. RNase inhibitors were omitted from the experiment and all washes after buffer 1 performed at room temperature (RT). The amounts of RNases A and T1 used to treat the elution fractions were reduced to 1/500 compared to the original protocol. A commented version of the protocol is available (Kilchert et al. 2020). We performed two sets of triplicate experiments (WT1 + *mtl1-1*; WT2 + *rrp6Δ* + *dis3-54*). WT1 and 2 data sets were merged for analysis of the WT interactome, and comparative interactomes were analyzed relative to the corresponding WT. A noCL control was included in the first experiment (WT1 + *mtl1-1*). Preparation of the samples for MS is described in the Supplemental Methods.

### Statistical data analysis

Background MS intensities were imputed at 18 (log_2_) for missing values at the level of the triplicate experiments before the data sets were merged. For the noCL-normalized interactome, raw MS intensities were used as provided in Supplemental Table S7. For WCE-normalized interactomes, raw MS intensities were normalized to median = zero for each data set before enrichment was calculated (Supplemental Table S8). Analysis was carried out in R (R Core Team 2019), and the code is provided for reference (Supplemental Code). For all protein data, relative differences were tested against zero with a moderated *t*-test using the limma package (eBayes function), release 3.32.10, to generate *P*-values (Ritchie et al. 2015). *P*-values adjusted for multiple testing (*Q*-values) were generated with p.adjust (method = “fdr” or “bonferroni”). Background values were imputed for WCE normalization if no MS intensities were available (open circles). If GO term or Pfam ID annotations are shown, proteins that were not detected in RIC were also included, as long as they were detected in the WCE; for these, background values were imputed for the interactome signal (crosses). For comparative RIC, we estimated the error for WT and mutant RIC/WCE ratios with uncertainty propagation using the propagate package and second-order Taylor expansion with the errors of mean RIC and mean WCE signals as input. For statistical analysis with limma, we simulated data with the exact statistics from the error propagation with mvrnorm (*n* = 3, empirical = TRUE) from the MASS package and tested relative differences against zero. Mutant RIC/WCE ratios were corrected by a constant factor to minimize global variance for all crosslinked proteins between WT and mutant before calculating fold-change values. GO enrichment analysis was performed with the enrichment analysis tool on the Gene Ontology Consortium server (release 20181018) (Ashburner et al. 2000; Mi et al. 2017). GO term and Pfam annotations in *S. pombe* were retrieved from EnsemblFungi using the biomaRt package in R, release 2.32.1 (Durinck et al. 2009), and information on individual proteins was accessed via PomBase (Lock et al. 2019). Plots were generated with the ggplot2 package (Wickham 2016).

### Crosslinking and immunoprecipitation (CLIP)

*S. pombe* cells were grown, 4sU-labeled, crosslinked, and lysed as for RIC. To validate RNA binding, partial CLIP was performed as described (König et al. 2010). In brief, cell lysates were diluted using two volumes of RQ1-buffer, and incubated with 2 units/mL Turbo DNase (Ambion) and 0.5 mg/mL RNase A (Qiagen) for 3 min at 37°C. Immunoprecipitation was performed with Anti-FLAG M2 Magnetic Beads (Sigma-Aldrich) for 2 h at 4°C. After washing with a buffer containing 1 M NaCl, RNA–protein complexes were radiolabeled with [γ-^32^P]-ATP and T4 polynucleotide kinase (New England Biolabs), resolved on SDS-PAGE, and transferred to nitrocellulose membrane. RNA-bound proteins were detected by autoradiography, and total immunoprecipitated protein subsequently analyzed by anti-FLAG western blot.

### Expression and purification of Cdk9/Pch1

*S. pombe* Pch1 (with an N-terminal His-tag followed by a TEV cleavage site) and Cdk9 were cloned into pACE-BacI vectors. Bacmids were generated in DH10EMBacY cells (Berger et al. 2004), then transfected into Sf9 cells (Thermo Fisher Scientific) grown in Insect-XPRESS (Lonza) with FuGENE HD transfection reagent (Promega) to generate V_0_ virus, which was harvested 120 h after transfection. V_1_ virus was produced by infecting 50 mL Sf9 cells grown at 27°C, 300 rpm with V_0_ virus (2E6 cell/mL, 1:100 [v/v] virus:cells). V_1_ viruses were harvested 72 h after proliferation arrest and stored at 4°C. For coexpression of Cdk9 and Pch1, 500 mL Sf9 cells (2E6/mL) were coinfected with both viruses (0.5:100 [v/v] each), cultivated for 72 h at 27°C and collected by centrifugation. Cells were harvested (238*g*, 10 min, 4°C), washed in PBS and snap-frozen in liquid N_2_. All subsequent steps were performed in the cold. Cells were lysed by sonication in WB buffer (25 mM Tris–HCl pH 8.0, 500 mM NaCl, 10 mM imidazole, 10 mM 2-Mercaptoethanol) freshly supplemented with EDTA-free protease inhibitor cocktail tablets (Roche) and 2500 units SuperNuclease (Sino Biological). The lysate was clarified by centrifugation at 230,000*g* for 40 min, filtered through a 0.45 μm filter, and applied to Ni-NTA resin (2 mL). After a wash with 30 mL WB buffer, proteins were eluted with 250 mM imidazole. The His-tag was cleaved with AcTEV protease (Thermo Fisher Scientific) at 4°C overnight, and proteins were run through a Superdex 200 (10/300) gel filtration column (GE Healthcare) equilibrated in buffer CB (20 mM HEPES 7.5, 150 mM NaCl, 0.5 mM MgCl_2_, 0.5 mM Mg(OAc)_2_, 1 mM 2-Mercaptoethanol), snap-frozen in liquid N_2_, and stored at −80°C.

### Cdk9/Pch1 kinase activity assays

Purified *S. pombe* Spt4/5 (Kecman et al. 2018) was used as kinase substrate. End-point reactions were carried out in 40 µL transcription buffer (25 mM HEPES pH 7.5, 40 mM NaCl, 5% [v/v] glycerol, 80 mM KCl, 0.2 mM DTT, 0.25 mM 2-Mercaptoethanol, 0.4 mM MnCl_2_, 0.5 mM MgCl_2_, 0.5 Mg(OAc)_2_, 0.51 mM ATP, 10 µM GTP, 1 µM UTP, 5 ng/µL dsDNA template, 0.5 units/µL of RNasin [Promega]), and 3.4 µg of Spt4/5 were incubated with 0.3 µg of Cdk9/Pch1 or buffer for 15 min at RT. After boiling in Laemmli buffer, 1 µg were run on a 7.5% acrylamide gel with 7.5 µM phospho-tag and 50 µM MnCl_2_. For the time-course experiment, Cdk9/Pch1 (40 pmol) was equilibrated in 20 mM HEPES pH 7.5, 30 mM NaCl, 1 mM DTT, 5% (v/v) glycerol, 0.01% (v/v) NP-40 and 1 mM MgCl_2_ in a total volume of 30 µL for 20 min at RT. In vitro kinase assays were performed in 60 μL reactions with 30 pmol Spt4/5 substrate. MnCl_2_, DTT, and [γ-^32^P]-ATP were added to a final concentration of 2.5 mM, 1 mM, and 1 μCi., respectively, and reactions incubated at RT. Ten microliters was taken for each time point. The reactions were stopped with LDS-loading buffer and heated for 5 min at 95°C before SDS-PAGE (4%–12%).

### Fluorescence anisotropy assays with Cdk9/Pch1

Fluorescence anisotropy experiments were carried out at RT with 8 nM FAM-labeled RNA of the sequence AUUAGUAAAAUAUAUGCAUAAAGACCAGGC (IDT). The RNA was heated for 5 min at 95°C before incubation with proteins. Titration of Cdk9/Pch1 was performed by serial dilution with CB buffer (20 mM HEPES 7.5, 150 mM NaCl, 0.5 mM MgCl_2_, 0.5 mM Mg(OAc)_2_, 1 mM 2-Mercaptoethanol) from 1240 nM to 0.32 nM and incubating with RNA substrate for 20 min at RT. The ligand was excited with linearly polarized light at 485 nm, and emission was measured at 520 nm in parallel and perpendicular planes to the excitation plane at 25°C using a FLUOstar-Omega microplate reader (BMG-Labtech). Each data point is an average of four readings from two different experiments. Anisotropy data were fitted with SigmaPlot using a standard four-parameter logistic equation to identify *K*_*d*_ as follows:

$$y=y_{\min}+\frac{y_{\max}-y_{\min}}{1+{\left(\frac{x}{K_{d}}\right)}^{-n}}$$

where *y*_min_ and *y*_max_ are the minimum and maximum anisotropy values, *x* represents the protein concentration, and *n* represents the Hill slope.

## Data access

The proteomics data generated in this study have been submitted to the ProteomeXchange Consortium (http://proteomecentral.proteomexchange.org) (Vizcaíno et al. 2014) under accession number PXD016741.

## Competing interest statement

The authors declare no competing interests.

## Supplementary Material

Supplemental Material
