# Supplementary material for: System-wide analyses of the fission yeast poly(A)+ RNA interactome reveal insights into organization and function of RNA–protein complexes
Source: Genome Res. 2020 Jul;30(7):1012–26. doi: 10.1101/gr.257006.119 (PMC7397868; doi:10.1101/gr.257006.119)
Supplement: Supplemental Material [file supp_30_7_1012__index.html]

System-wide analyses of the fission yeast poly(A)+ RNA interactome reveal insights into organization and function of RNA–protein complexes — System-wide analyses of the fission yeast poly(A)+ RNA interactome reveal insights into organization and function of RNA–protein complexes — Supplemental Material 

# System-wide analyses of the fission yeast poly(A)+ RNA interactome reveal insights into organization and function of RNA–protein complexes

## Supplemental Material

- Supplemental\_Figures.pdf
- Supplemental\_Methods.pdf
- Supplemental\_Table\_S1.xlsx
- Supplemental\_Table\_S2.xlsx
- Supplemental\_Table\_S3.xlsx
- Supplemental\_Table\_S4.xlsx
- Supplemental\_Table\_S5.xlsx
- Supplemental\_Table\_S6.xlsx
- Supplemental\_Table\_S7.xlsx
- Supplemental\_Table\_S8.xlsx
- Supplemental\_Code.R
