# Supplementary material for: System-wide analyses of the fission yeast poly(A)+ RNA interactome reveal insights into organization and function of RNA–protein complexes
Source: Genome Res. 2020 Jul;30(7):1012–26. doi: 10.1101/gr.257006.119 (PMC7397868; doi:10.1101/gr.257006.119)
Supplement: Supplemental Material [file supp_gr.257006.119_Supplemental_Figures.pdf]

Kilchert et al., Supplemental Figure 1

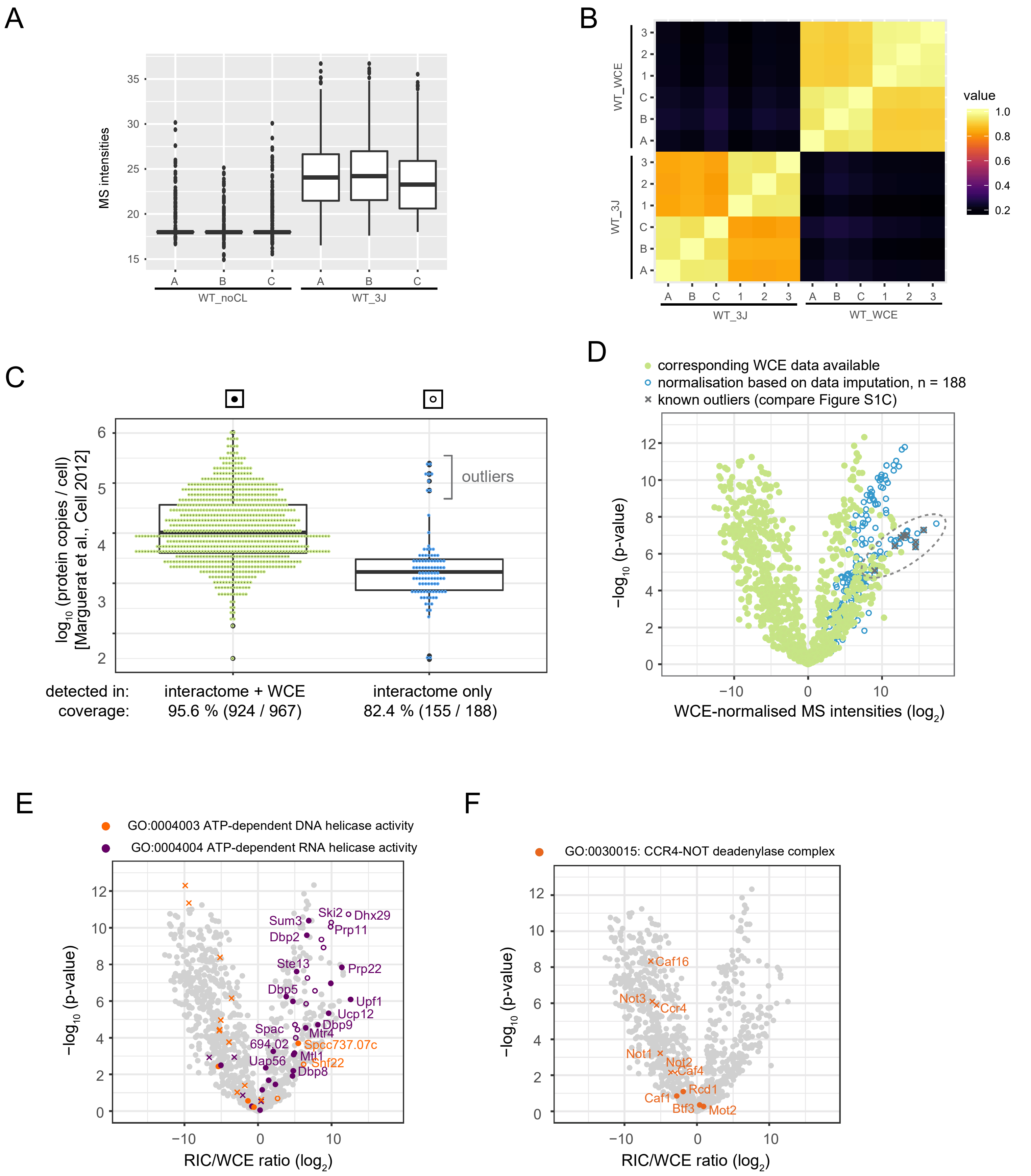

Figure S1

(**A**) Boxplot of raw MS intensities (log_2) of proteins recovered from oligo-d(T) pull-downs of UV-crosslinked samples (3 J/cm^2) versus non-irradiated controls (noCL) (n=3). Few proteins were detected in the noCL control; for proteins without signal, background values were imputed to be able to calculate RIC enrichment. The lower and upper hinges correspond to the first and third quartiles (the 25th and 75th percentiles). The bold line indicates the mean of the data. The lower and upper whiskers extend from the hinge to the smallest and largest value no further than 1.5 * IQR from the hinge, respectively, where IQR is the interquartile range. (**B**) Spearman's correlation plot comparing raw MS intensities (log_2) of proteins recovered from oligo-d(T) pull-downs of UV-crosslinked samples (3 J/cm^2) and the respective whole cell extracts (WCE) (n = 6). (**C**) Cellular copy numbers for crosslinked proteins that either were (left, green) or were not (right, blue) detected in the corresponding WCE samples; copy numbers are taken from Marguerat et al. (2012). Coverage designates the fraction of proteins for which copy numbers were available in the Marguerat et al. (2012) data set. On average, cellular copy numbers of proteins that could not be detected in the WCE samples were low compared to proteins that were detected, providing justification for the imputation of background values. Outliers are marked – these are Rpl1201, Hhf2, Eft202, Rps1801, Rps1401, Rps1101, Rps2302, and Rpl1102, and predominantly correspond to ribosomal proteins. 33 crosslinked proteins without WCE signal were not present in the Marguerat et al. (2012) data set and could not be included in the graph. (**D**) Volcano plot, p-values (-log, moderated Student's t-test) are plotted against the fold change of the mean MS intensities (log_2) of proteins recovered from the oligo-d(T) pull-downs of UV-crosslinked samples (3 J/cm^2) over the input WCE (n=6). Crosslinked proteins that either were (green, full circles) or were not (blue, empty circles) detected in the corresponding WCE input samples are indicated. Among proteins without WCE signal, outliers with high cellular copy numbers according to Marguerat et al. (2012) (compare S1C) are designated with crosses. We regard proteins without WCE signal that fall in the region of the plot where these outliers cluster (marked with an ellipse) as low confidence data points. Most low confidence data points correspond to ribosomal proteins (compare also S2A). (**E, F**) Distribution of proteins annotated with various GO terms in the WCE-normalised RNA interactome. Volcano plots as in 1D. Full circles denote proteins that were detected in both oligo-d(T) pull-down and WCE, empty circles proteins that were present in the oligo-d(T) pull-downs but not the WCE, crosses proteins that were detected in the WCE, but never in the oligo-d(T) pull-downs (see also 1C). In (**E**), proteins annotated with GO function "ATP-dependent DNA helicase activity" [GO:0004003] or "ATP-dependent RNA helicase activity" [GO:0004004] are indicated in orange and purple, respectively. While annotated RNA helicases showed high average enrichment in RIC, annotated DNA helicases rarely crosslinked; only two proteins, Spcc737.07c and Snf22, were significantly enriched, possibly indicating dual specificity.

Kilchert et al., Supplemental Figure 2

A

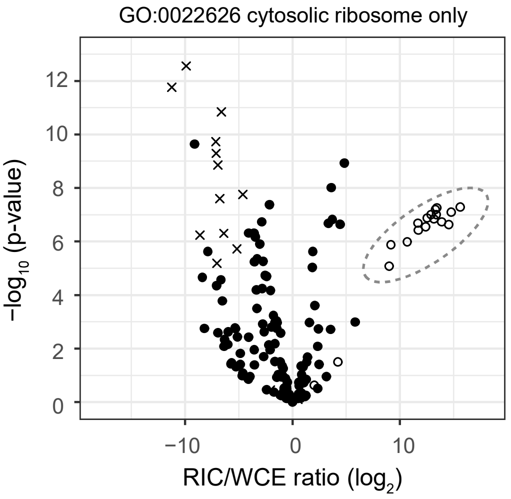

B

Gene standard name	Product
rps2	40S ribosomal protein S2
rps3	40S ribosomal protein S3
rps1002	40S ribosomal protein S10
rps2601	40S ribosomal protein S26
rps2602	40S ribosomal protein S26
rps502	40S ribosomal protein S5

Figure S2

(**A**) Distribution of ribosomal proteins ("cytosolic ribosome", [GO:0022626]) in the WCE-normalised RNA interactome. Volcano plot, p-values (-log, moderated Student's t-test) are plotted against the fold change of the mean MS intensities (log_2) of proteins recovered from the oligo-d(T) pull-downs of UV-crosslinked samples (3 J/cm^2) over the input WCE (n=6). Full circles denote proteins that were detected in both oligo-d(T) pull-down and WCE, empty circles proteins that were present in the oligo-d(T) pull-downs but not the WCE, crosses proteins that were detected in the WCE, but never in the oligo-d(T) pull-downs. RPs with imputed WCE values were among the low-confidence data points (compare Figure S1C and D), and were disregarded in the subsequent analysis. (**B**) Standard gene names and names of protein products for RPs enriched in poly(A)$^+$ RIC.

Kilchert et al., Supplemental Figure 3

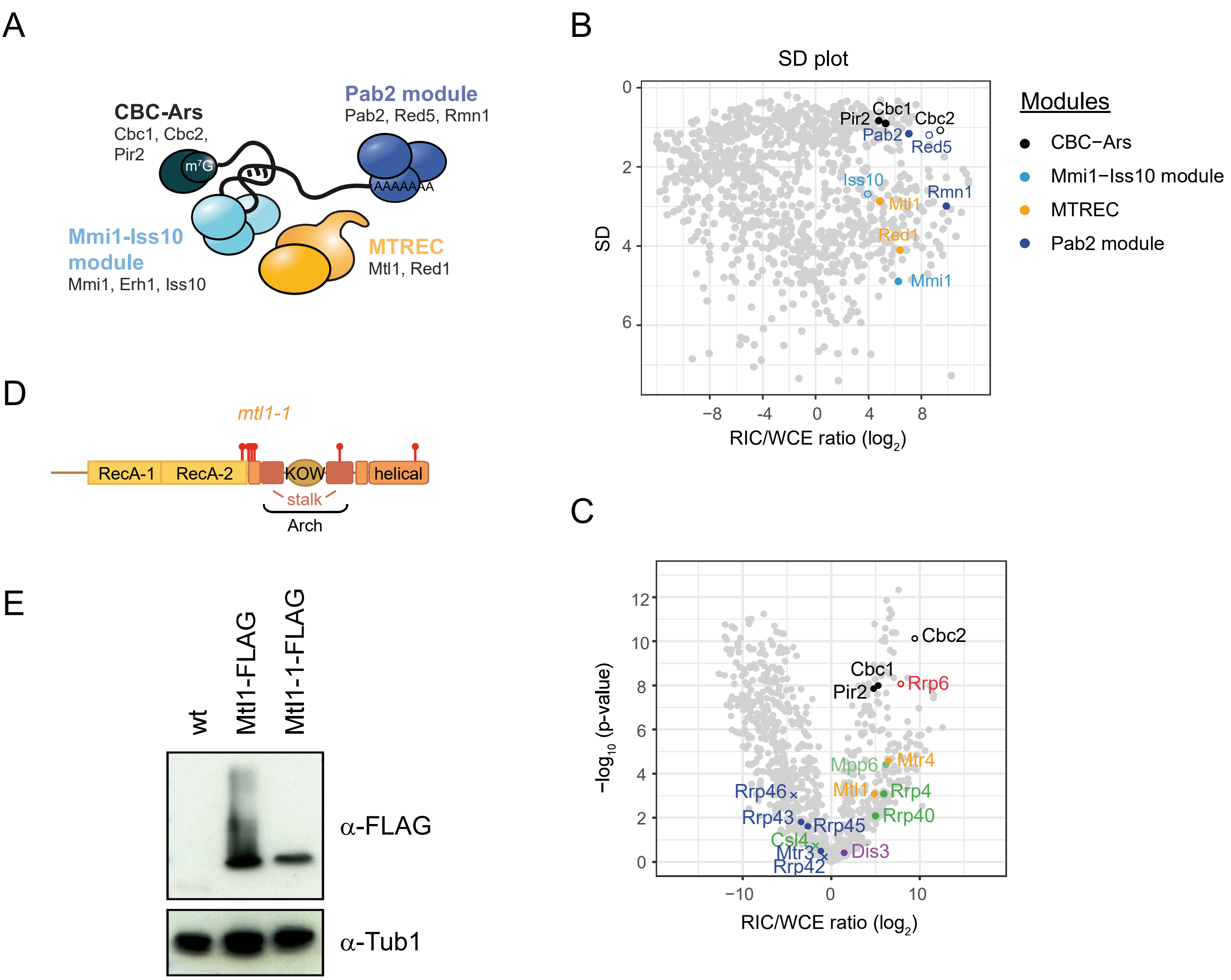

Figure S3

(**A**) Co-factors of the nuclear RNA exosome are organised into modules. Schematics based on (Egan et al., 2014; Lee et al., 2013; Shichino et al., 2018; Zhou et al., 2015) (**B**) SD plot of the WCE-normalised poly(A)+ RNA interactome with co-factors of the nuclear exosome highlighted. Colour scheme as in (A). Erh1 was not detected in the RIC experiment. (**C**) Volcano plot of the WCE-normalised poly(A)+ RNA interactome. Components of the nuclear exosome are highlighted. Full circles denote proteins that were detected in both oligo-d(T) pull-down and WCE, empty circles proteins that were present in the oligo-d(T) pull-downs but not the WCE, crosses proteins that were detected in the WCE, but never in the oligo-d(T) pull-downs (see also 1C). (**D**) Domain organisation of Mtl1. Positions of the point mutations the *mtl1-1* mutant are marked as lollipops. (**E**) The mutant Mtl1-1 protein is expressed at similar levels to the wild-type protein; however, the band for the mutant protein is more defined, suggesting that some posttranslational modification may be lost. Equal amounts of yeast lysate were loaded onto SDS-PAGE and analysed by Western blot for FLAG. An untagged strain was included for reference. Tubulin was used as loading control.

Kilchert et al., Supplemental Figure 4

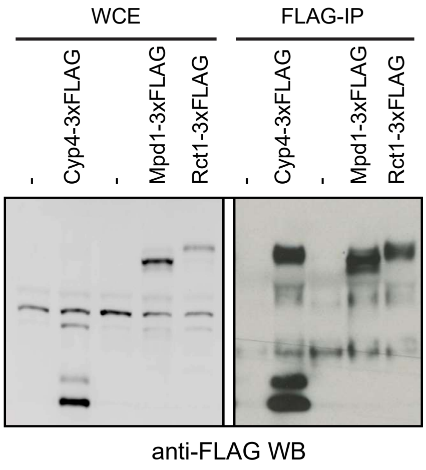

Figure S4

Because the high apparent molecular weight species of Cyp4 migrates very close to Rct1, we performed test-IPs from the WCEs and control strains to exclude that we were looking at an IP contaminant. FLAG immunoprecipitations (IP) from whole cell extracts (WCE) of 4sU-labelled crosslinked cells (3J / cm^2) used for CLIP and control strains, untagged and tagged (Mpd1-3HF).

Kilchert et al., Supplemental Figure 5

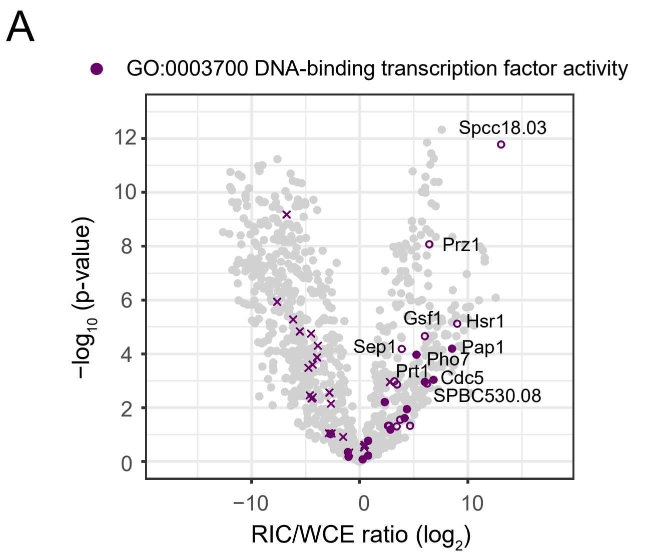

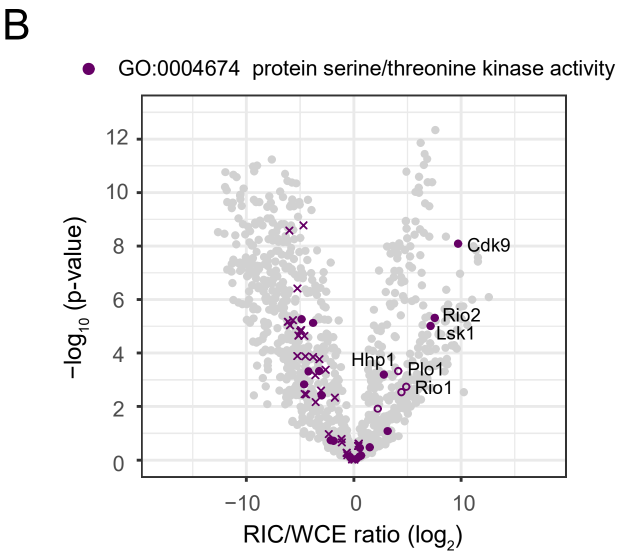

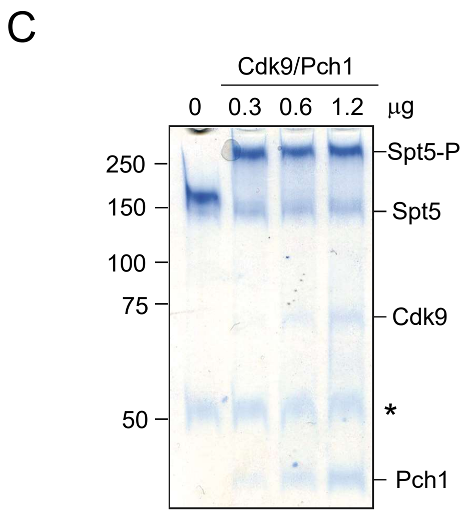

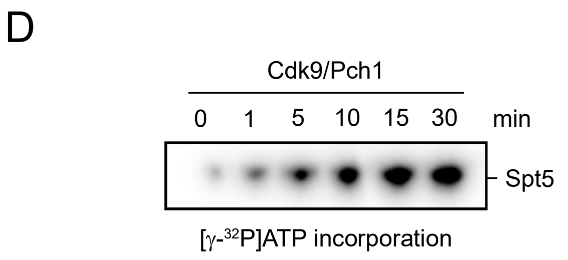

Figure S5

(**A**, **B**) Distribution of proteins annotated with various GO terms in the WCE-normalised RNA interactome. Volcano plot, p-values (-log, moderated Student's t-test) are plotted against the fold change of the mean MS intensities (log_2) of proteins recovered from the oligo-d(T) pull-downs of UV-crosslinked samples (3 J/cm^2) over the input WCE (right panel) (n=6). Full circles denote proteins that were detected in both oligo-d(T) pull-down and WCE, empty circles proteins that were present in the oligo-d(T) pull-downs but not the WCE, crosses proteins that were detected in the WCE, but never in the oligo-d(T) pull-downs (see also 1C). (**C**) Phospho-tag gel of *in vitro* kinase assays with increasing amounts of Cdk9/Pch1 on recombinantly purified Spt4/5. Phospho-tag changes the migration of phosphorylated proteins. The asterisk marks an unspecific band that copurifies with Spt5. (**D**) Autoradiogram of *in vitro* kinase assays of Cdk9/Pch1 with [γ-^{32}P]ATP on recombinantly purified Spt4/5.
