# Supplementary material for: System-wide analyses of the fission yeast poly(A)+ RNA interactome reveal insights into organization and function of RNA–protein complexes
Source: Genome Res. 2020 Jul;30(7):1012–26. doi: 10.1101/gr.257006.119 (PMC7397868; doi:10.1101/gr.257006.119)
Supplement: Supplemental Material [file supp_gr.257006.119_Supplemental_Methods.pdf]

Supplemental Methods

Sample preparation for mass spectrometry and data acquisition

Samples were loaded onto Vivacon500 filters and denatured in 8 M urea, 100 mM TEAB for 30 min. TCEP was added to a final concentration of 10 mM and the sample reduced for 30 min, then alkylated with 50 mM CAA (final concentration) for 30 min in the dark. The filters were washed with 6 M urea, 50 mM TEAB until the complete removal of detergent. Proteins were digested on filter with 1 µg LysC in 6 M urea, 50 mM TEAB for 4h at 37˚C, then with 1 µg trypsin overnight. The flow-through was collected, the filters washed with 200 µl 0.1% TFA, then with 200 µl 50% ACN, 0.1% TFA, all flow-through fractions pooled and dried in a centrifugal evaporator. Samples were resuspended in 50 µL 5% DMSO, 5% FA and analysed on an Ultimate 3000 nanoUHPLC system (Thermo Fisher Scientific) coupled to a QExactive mass spectrometer (Thermo Fisher Scientific). The LC was configured to contain a C18 PepMap100 pre-column (300 µm i.d. x 5 mm, 100 Å, Thermo Fisher Scientific) and an in-house packed analytical column (50 cm x 75 µm i.d. packed with ReproSil-Pur 120 C18-AQ, 1.9 µm, 120 Å) using a 2 h linear gradient (7% to 28% solvent B (0.1% FA in ACN), flow rate: 200 nL/minute). The raw data was acquired in a data-dependent mode (DDA). Full scan MS spectra were acquired in the Orbitrap (scan range 350-1500 m/z, resolution 70,000, AGC target 3xe6, maximum injection time 100 ms), and the 20 most intense peaks selected for HCD fragmentation at 30% of normalised collision energy. HCD spectra were also acquired in the Orbitrap (resolution 17,500, AGC target 5xe4, maximum injection time 120 ms) with first fixed mass at 180 m/z. The acquired QExactive Raw MS data were processed with MaxQuant (Tyanova et al., 2016). Data were searched against the UniProt *S. pombe* database alongside a list of common contaminants provided by the software. The search parameters for the Andromeda search engine within MaxQuant were: full tryptic specificity, allowing two missed cleavage sites, fixed modification was set to carbamidomethyl (C) and the variable modification to acetylation (protein N-terminus), oxidation (M). Match between runs was applied. All other settings were set to default leading to a protein false discovery rate (FDR) of 0.01.

Supplemental References

Tyanova, S., Temu, T., and Cox, J. (2016). The MaxQuant computational platform for mass spectrometry-based shotgun proteomics. Nat. Protoc. *11*, 2301–2319.
