# Supplementary material for: System-wide analyses of the fission yeast poly(A)+ RNA interactome reveal insights into organization and function of RNA–protein complexes
Source: Genome Res. 2020 Jul;30(7):1012–26. doi: 10.1101/gr.257006.119 (PMC7397868; doi:10.1101/gr.257006.119)
Supplement: Supplemental Material [file supp_gr.257006.119_Supplemental_Code.R.html]

Supplemental\_Code 

# System-wide analyses of the fission yeast poly(A)+ RNA interactome reveal insights into organization and function of RNA–protein complexes
